# Supplementary material for: The Association between Cyclin Dependent Kinase 2 Associated Protein 1 (CDK2AP1) and Molecular Subtypes of Lethal Prostate Cancer
Source: Int J Mol Sci. 2022 Nov 1;23(21):13326. doi: 10.3390/ijms232113326 (PMC9658869; doi:10.3390/ijms232113326)
Supplement: Supplementary file 1 [file ijms-23-13326-s001.zip › ijms-1958269-supplementary.pdf]

International Journal of Molecular Sciences

MDPI

Supplementary Table S1. CDK2AP1 expression in metastatic and non metastatic PCa.

Variables	No Mets	Mets	p-value
**CDK2AP1**			
Score 0 or 1	119 (51.5)	15 (34.1)	0.034
Score 2 or 3	112 (48.5)	29 (65.9)	
**AR**			
Score 0 or 1	15 (6.7)	7 (17.9)	0.019
Score 2 or 3	209 (93.3)	32 (82.1)	
**PTEN**			
Score 0	74 (32.7)	19 (45.2)	0.118
Score 1,2,3	152 (67.3)	23 (54.8)	
**ERG**			
Negative	169 (74.8)	30 (69.8)	0.492
Positive	57 (25.2)	13 (30.2)	
**P53**			
Score 0,2,3	38 (18.4)	11 (28.2)	0.158
Score 1	169 (81.6)	28 (71.8)	
**CDK2AP1/PTEN**			
HR PTEN + HR CDK2AP1	51 (22.6)	15 (34.9)	0.080
HR PTEN + LR CDK2AP1	23 (10.2)	5 (11.6)	
LR PTEN + HR CDK2AP1	59 (26.1)	14 (32.6)	
LR PTEN + LR CDK2AP1	93 (41.2)	9 (20.9)	
**CDK2AP1/AR**			
HR AR + HR CDK2AP1	7 (3.1)	3 (7.7)	0.013
HR AR + LR CDK2AP1	8 (3.6)	4 (10.3)	
LR AR + HR CDK2AP1	103 (46.0)	23 (59.0)	
LR AR + LR CDK2AP1	106 (47.3)	9 (23.1)	
**CDK2AP1/ERG**			
HR ERG + HR CDK2AP1	40 (17.7)	10 (23.3)	0.141
HR ERG + LR CDK2AP1	17 (7.5)	3 (7.0)	
LR ERG + HR CDK2AP1	70 (31.0)	19 (44.2)	
LR ERG + LR CDK2AP1	99 (43.8)	11 (25.6)	
**CDK2AP1/p53**			
HR p53 + HR CDK2AP1	29 (14.0)	10 (25.6)	0.061
HR p53 + LR CDK2AP1	9 (4.3)	1 (2.6)	
LR p53 + HR CDK2AP1	79 (38.2)	19 (48.7)	
LR p53 + LR CDK2AP1	90 (43.5)	9 (23.1)	

HR (high risk) PTEN; PTEN negative, LR (low risk) PTEN; PTEN weak, moderate and high intensity

HR ERG; ERG positive, LR ERG, ERG negative

HR CDK2AP1; negative and weak intensity

LR CDK2AP1; moderate and high intensity

HR p53 (p53; 0), LR p53 (p53; 1,2,3) (see methods for further explanation).

Supplementary Table S2. Number of patients at risk

Timepoints/Categories	0 months	20 months	40 months	60 months	90 months	100 months
**CDK2AP1 and PTEN combined**						
High risk PTEN and High risk CDK2AP1	62	32	19	9	6	
High risk PTEN and Low risk CDK2AP1	28	17	10	3		
Low risk PTEN and High risk CDK2AP1	72	55	35	11	3	
Low risk PTEN and Low risk CDK2AP1	98	85	63	37	7	1
**CDK2AP1 and ERG combined**						
High risk ERG and High risk CDK2AP1	48	31	17	8	5	
High risk ERG and Low risk CDK2AP1	20	13	8	2		
Low risk ERG and High risk CDK2AP1	86	56	37	12	4	
Low risk ERG and Low risk CDK2AP1	106	89	65	38	7	1
**CDK and P53 combined**						
High risk P53 and High risk CDK2AP1	27	13	7	3	2	
High risk P53 and Low risk CDK2AP1	9	4	4	**1**		
Low risk P53 and High risk CDK2AP1	94	73	46	16	7	
Low risk P53 and Low risk CDK2AP1	96	82	58	32	5	1

Supplementary Table S3. Hydrogen Bonding Contacts

CDK2AP1 Residue	p53 residue(s)
G2	E198
H3	H233
H8	E224
H10	Q144, S227
S11	D228
E21	H115
E22	L111
K25	E286
E26	N131
S36	Q100
E39	K101-T102
R40	N268
G44	R110

Supplementary Table S4. Physical contacts within 4 angstroms

CDK2AP1 Residue	p53 residue(s)
M1	D186, R196, S185, D186, N235, E198
G2	E198, G199,
H3	G199, N200, L201, H233,
H4	T140, E198,
H5	H233
H6	T140, P142, H233, T231,
H8	E224, V225, G226
S9	G226
H10	Q144, W146, C229, D228, G226
S11	D228
E15	W146,
L17	H115
E21	H115, P128
E22	L111, Q104,
K25	P128, E286, R282, S127, P128, A129
E26	N131,
R28	L130
S36	Q100. K101, T102
E39	K101, T102
R40	Q104, S269, L111, N268, N131, T102,
R43	Q1O4, S106, G108, D148, R110,
G44	R110
H47	D148, R110
